# Mental health in children conceived by Assisted Reproductive Technologies (ARTs): Insights from a longitudinal study of Australian children

**DOI:** 10.1371/journal.pone.0304213

**Published:** 2024-06-27

**Authors:** Md. Irteja Islam, Oscar A. Chaffey, Verity Chadwick, Alexandra Martiniuk

**Affiliations:** 1 Sydney School of Public Health, Faculty of Medicine and Health, The University of Sydney, Camperdown, New South Wales, Australia; 2 Research, Innovation and Grants, Spreeha Bangladesh, Dhaka, Bangladesh; 3 Centre for Health Research, The University of Southern Queensland, Darling Heights, Queensland, Australia; 4 Obstetrics and Gynaecology Department, Royal Prince Alfred Hospital, Camperdown, New South Wales, Australia; 5 The George Institute for Global Health, Newtown, New South Wales, Australia; 6 Dalla Lana School of Public Health, The University of Toronto, Toronto, Ontario, Canada; University of Melbourne, AUSTRALIA

## Abstract

**Background:**

The mental health of children conceived using Assisted Reproductive Technologies (ARTs) such as In-Vitro-Fertilization (IVF) is a subject of significant controversy. Existing studies suggest children conceived through ART meet physical and cognitive developmental milestones at similar rates to their spontaneously conceived peers, however, a significant number of studies have connected ART conception with mental health conditions, particularly depression and attention-deficit hyperactivity disorder (ADHD) in adolescence. This study, therefore, aimed to determine whether maternal use of ARTs to achieve pregnancy is associated with an increased risk of mental disorders in these children, and whether these effects are sex-dependent or confounded by known covariates in the ART population.

**Methods:**

Secondary data analysis was performed using Growing Up in Australia: The Longitudinal Study of Australian Children (LSAC) data; a nationally representative population-based cross-sequential cohort study. Multivariate logistic regression models examined the impact of ART (including IVF and other fertility drugs, from LSAC wave-1 and wave-2 conducted in 2004 and 2006, respectively) on mental health outcomes (i.e., autism, ADHD, anxiety and/or depression, from LSAC waves 8 conducted in 2018) in Australian adolescents aged 18–19 years in 2018 (n = 1735). Known sociological and obstetric covariates including maternal age, birth weight, smoking and drinking alcohol during pregnancy, maternal gestational diabetes, postnatal depression, hypertension, and socioeconomic status were considered to generate an adjusted logistic model. Variables with a p-value of <0.05 in the regression models were considered statistically significant.

**Results:**

Of the 1735 mother-child dyads analysed, the maternal mean age was 35.6 years (Standard deviation = ±4.75), approximately 5% of mothers (n = 89) used ART to become pregnant, and 22% of adolescents (n = 384) had a mental disorder. Longitudinal analysis revealed no relationship between ART and children developing a mental disorder in the LSAC population.

**Conclusion:**

These results should reassure parents considering ART that there is no increased risk of psychological or neurodevelopmental problems in their ART conceived offspring.

## Introduction

Infertility is one of the most common reproductive health concerns, impacting a large proportion of the population worldwide [1, 2]. Globally, the rate of infertility varies substantially due to demographic, cultural, and genetic factors as well as access to health care [3]. It is estimated that around 10–15% of couples of reproductive ages are affected by infertility. Increased access to health care and reproductive technologies, as well as delayed child bearing in some countries, has resulted in an increasing number of couples using assisted reproductive technologies (ARTs) to conceive [4, 5]. In the last two decades, ART use has nearly doubled [6]. In Australia, 4.9% of women who gave birth in 2019 used some form of assisted reproductive technology and the use of these technologies increased by 6.2% between 2018 and 2019 [5, 6]. The most common and most well-studied method of ART is known as in vitro fertilisation (IVF), where oocytes are fertilised extracorporeally and inseminated using *in vitro* insemination or through intracytoplasmic sperm injection [5]. One of the main indications is older maternal age; the average age at pregnancy for women using IVF is higher than for women who spontaneously conceive; in Australia of couples using ART the average maternal age is 35.8 years, and paternal age is 38 years in 2019 [7, 8].

The average IVF “cycle” in Australia has a 23.2% success rate at producing a clinical pregnancy and an 18.3% chance of an eventual live birth, although this varies considerably with maternal age [8]. In women under 30 who use ART, the live birth rate in 2019 was substantially higher, at 40.4% [7]. The general health and development of children conceived through IVF has attracted significant research attention and the results have been encouraging. Reviews in 2014 and 2022 found that the growth, development and cognitive function of children conceived through IVF are comparable to that of spontaneously conceived children [9, 10]. However evidence demonstrates that children born using IVF are, however, at significant increased risk of preterm birth and low birthweight, as well as congenital cardiac, musculoskeletal and genitourinary malformations [11, 12]. Any research results need to be considered for potential confounding by the underlying subfertility and advanced age of ART users: for instance, advanced maternal age is associated with congenital malformations and chromosomal disorders independent of ART use [13, 14]. Further, evidence suggests that children conceived through IVF are epigenetically comparable to their spontaneously conceived peers and any differences between these groups are thought to normalize by adulthood [15, 16].

Moreover, psychiatric problems in all children are common: 10% of children will have a significant mental health problem that impacts their day-to-day life at some point in their childhood [17]. These problems are also overrepresented in Australia relative to the global population perhaps due to a relatively strong health system increasing the detection and recording of mental illness [18]. As a result, establishing the relationship between IVF conception and psychiatric outcomes in childhood and adolescence is a subject of considerable interest. In a 2011 review, children conceived through IVF were found to have broadly similar psychological experiences and degrees of social adjustment to those of their spontaneously conceived peers [19]. Multiple recent studies have also found that children conceived through IVF had comparable levels of well-being in adulthood to those conceived spontaneously [20, 21]. Two studies even found that children born through IVF tended to perform better in school, although this effect was confounded in one study by the higher socioeconomic status of parents who used IVF [22, 23].

Despite these encouraging results, there has still been some cause for concern. Multiple studies have reported that children conceived through IVF are at an increased risk of neurodevelopmental disorders such as cerebral palsy, intellectual disability, tic disorders and autism [24–27]. One 2005 study explained these results by suggesting that the risk could be “entirely explained’’ by the confounding effect of low birthweight in offspring born by IVF and that there was no additional risk to children conceived through IVF who were born at a healthy birthweight and full term [24]. Other studies have reported that the general risk of being diagnosed with any mental disorder is higher in children conceived through IVF than in spontaneously conceived counterparts [28–31]. Four studies have established a relationship between IVF conception and an increased risk of developing major depressive disorder (MDD) in childhood [27, 30–32]. A further three studies have suggested that IVF conception is a risk factor in developing ADHD, although the effect observed has been weak and stronger in girls [31–34].

This study will aim to contribute further data to three core uncertainties in the present literature by using a population-based cross-sequential cohort of Australian children. First, there is considerable controversy about whether the use of ART in mothers longitudinally impacts the mental health of their offspring in adolescence and/or early adulthood. This study will contribute additional data to the literature around this question from a robust population-based longitudinal cohort study. Secondly, it is presently unclear whether the postulated relationship between ART and mental health persists into adulthood; indeed, one study suggested a time-dependent relationship where the negative effect of IVF conception waned by late adolescence [32]. Only one study that found a connection between these variables studied children conceived through ART when they were adults; it was, however, a survey-based study that had not followed those children throughout their lives and was conducted in the 1990s [30]. The Longitudinal Study of Australian Children (LSAC) data, which will be used for this study, has followed ART children for over two decades and will allow further elucidation of the time dependency of these results. Finally, the impact of sociological and obstetric covariates including subfertility, parent age, preterm birth, birth weight, and socioeconomic status remains controversial. Some studies have suggested that these covariates entirely explain any deleterious impact of ART [24] whereas others have suggested that they are a statistically significant confounder that does not completely erase the proposed impact on mental health [30, 34]. Moreover, this study will aim to clarify these questions by generating a logistic regression model that accounts for these confounding variables on the mental health of Australian children born through ART.

## Methods

### Study design

This study gained access to data from the Growing Up in Australia: The Longitudinal Study of Australian Children (LSAC), a population-based cross-sequential cohort study. The LSAC was conducted by the Australian Government Department of Social Services (DSS) in partnership with the Australian Bureau of Statistics (ABS) and the Australian Institute of Family Studies (AIFS). The list of eligible participants for LSAC were obtained from an online enrolment database for the Australian universal healthcare insurance scheme, Medicare. LSAC used a multi-stage cluster sampling design where the representative postcodes were initially selected using the probability proportion to size sampling method, stratifying participants by state and then by urban and rural areas. Children were then randomly selected from 311 postcodes, with approximately 40 children per postcode in large states and 20 per postcode in small states.

LSAC has collected biennial data from two cohorts—the birth cohort (B cohort) who were 0–1 years old at baseline (n = 5107) and the kindergarten cohort (K cohort) who were 4–5 years old at baseline (n = 4983) in 2004. At baseline in LSAC wave 1, 10090 children were recruited, while 4188 responded in the latest wave (i.e. 9C2) which was collected by online survey during the COVID pandemic in between June and September 2021. Data was collected primarily from parents (typically the mother) with additional information given by other caregivers. Children over 10 years were asked to contribute to specific questions. Data collection methods prior to COVID-19 included face-to-face interviews, mail-out questionnaires, time-use diaries, Computer-Assisted Self-Interview and Web-Interview (CASI and CAWI). Due to the COVID-19 pandemic in 2020, only CAWI was used in LSAC waves 9C1 and 9C2. More details on the LSAC methodology, including sampling procedures and data collection techniques, are described elsewhere [35, 36].

**Fig 1:**
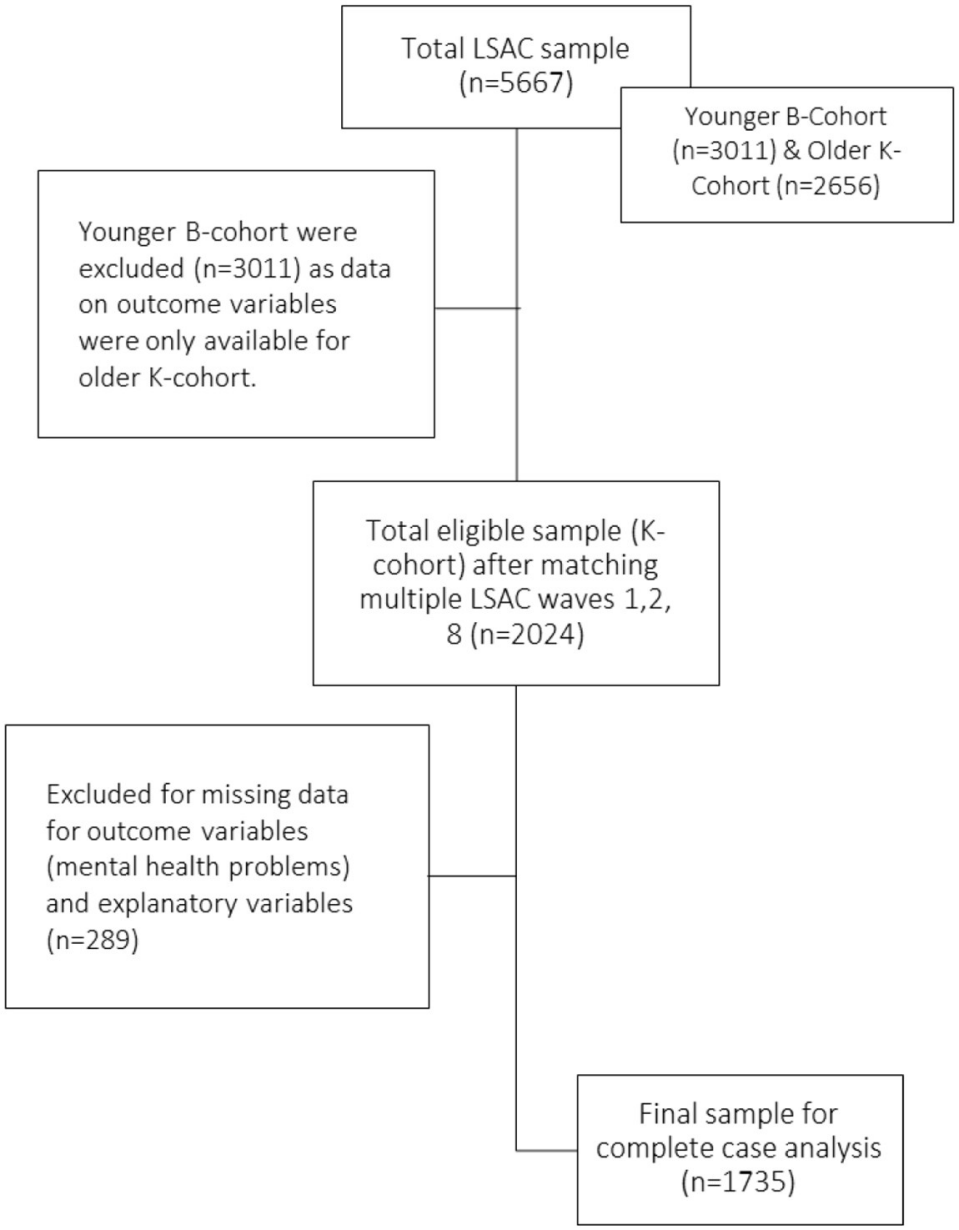
Flow chart for sample selection.

The current study included 1735 mother-child dyads—Mothers who reported using ARTs during pregnancy (Yes/No) in LSAC waves 1 and 2, respectively, in 2004 and 2006, were longitudinally matched with the study child who reported the presence of a mental disorder (Yes/No) in LSAC waves 8 in 2018. We included complete data on the outcome variable (mental disorders which were ascertained in the LSAC as including: autism, ADHD, anxiety and/or depression in children/adolescents)), main explanatory variable (history of using ARTs by mothers of the study child) and other covariates in our study. Participants who did not respond to outcome or predictor variables were omitted (n = 225). A flow chart for the selection of the analytical sample is presented in Fig 1.

### Measures

Variables associated with the history of using ARTs in mothers as well as mental disorders in children were included in this study (Table 1) [28, 29, 32, 34].

**Table 1:** List of variables.

Variables	Description of variables
*Outcome variable*	
Mental disorders	Whether the study child had been diagnosed with any mental disorders (including autism, ADHD, anxiety and/or depression). Response options are categorised as ’No’ (coded 0) and ’Yes’ (coded 1).
*Main Explanatory variable*	
Assisted reproductive technologies (ARTs)	Whether the biological mother of the study child used any ARTs such as IVF or any other fertility drugs for the pregnancy. Response options were ’No’ (coded 0) and ’Yes’ (coded 1).
*Covariates*	
Sex of the study child	The study child’s sex was categorized into ‘Boys’ (coded as 0) and ‘Girls’ (coded as 1).
Maternal age	Age was used as a continuous variable
Smoked during pregnancy	Whether the mother smoked during pregnancy—‘No’ (coded 0) and ‘Yes’ (coded 1).
Drank alcohol during pregnancy	Whether the mother of the study child consumed alcohol during pregnancy—‘No’ (coded 0) and ‘Yes’ (coded 1).
Diabetes during pregnancy	Whether the mother of the study child was diagnosed with diabetes during pregnancy—‘No’ (coded 0) and ‘Yes’ (coded 1).
Hypertension during pregnancy	Whether the mother of the study child was diagnosed with HTN during pregnancy—‘No’ (coded 0) and ‘Yes’ (coded 1).
Postnatal depression after pregnancy	Whether the mother of the study child was diagnosed with postnatal depression after pregnancy—‘No’ (coded 0) and ‘Yes’ (coded 1).
Mother’s employment status during pregnancy	Whether the mother of the study child worked during pregnancy—‘Employed’ (coded 0) and ‘Unemployed’ (coded 1).
Gestational age	According to the AIHW [7] and availability of data, we categorised gestational age into three: Term (37–41 weeks of pregnancy)—coded as 0, preterm (20–36 weeks of pregnancy)—coded as 1, and post-term pregnancy (>42 weeks)—coded as 2.
Study child’s birth weight	According to the AIHW [7] and availability of data, we categorised the child birthweight into two: normal (birthweight between 2500 and <4500 grams)—coded as 0, and low birth weight (birthweight <2500 grams)—coded as 1.
Area of residence	The Australian Statistical Geography Standard (ASGS) [37] classifies Remoteness Areas into five categories of relative remoteness across the country—Major Cities of Australia, Inner Regional Australia, Outer Regional Australia, Remote Australia, and Very Remote Australia. Within these categories, a binary variable of ’Area of Residence’ was generated. ’Major cities’ were coded as 0, and ’inner regional’, ’outer regional’, ’remote’ and ’very remote’ were combined to be a variable labelled ’regional/remote’ and coded as 1.
IRSAD quintiles	The Index of Relative Socio-economic Advantage and Disadvantage (IRSAD) [38] collates data about the economic and social conditions of people and households within an area, including both relative advantage and disadvantage measures. The lowest quintile (Quintile 1, 0–20%) signifies the greatest disadvantage and a lack of advantage in general. The highest quintile (Quintile 5, 80–100%) signifies the greatest advantage and relative lack of disadvantage.

### Statistical analysis

Initially, descriptive statistics (frequency (n) and percentages (%) for categorical variables; mean and standard deviation for continuous variables) were computed for the total sample population and the sample stratified by the history of ARTs usage by the study child’s mother. We then conducted a multivariate logistic regression analysis, factors associated with outcome variables in the unadjusted regression analysis, with a p-value of less than 0.05 considered statistically significant and adjusted in the logistic models. The results of the regression analyses were presented as odds ratios (OR) with corresponding 95% confidence intervals (CI). As recommended by the LSAC data user guide, we used the ‘SVY’ command of Stata/SE 14.1 to consider the LSAC’s complex survey design that included stratification, clustering, and weighting, and to deal with potential non-response bias and to avoid overestimation of statistical significance. Further, the Goodness-of-fit test was utilised to evaluate the assumptions of the regression model, the Variance Inflation Factor (VIF) test was employed to identify any multicollinearity among the predictor variables, and the Link test was employed to verify the logit model’s specification. Finally, the receiver operating characteristic (ROC) curve analysis was conducted as a post-hoc procedure to logistic regression to verify the predictive power.

### Ethics

The LSAC was approved by the Human Research Ethics Committee of the Australian Institute of Family Studies (AIFS) (Application number 20–09), and all study participants provided written informed consent. Furthermore, the authorship team secured authorisation to utilise LSAC data for research and publishing from the Australian Data Archive Dataverse (Application Reference No. 263493). Since we used a routinely collected and completely anonymous dataset and published the results in a non-identifiable format in compliance with the National Statement on Ethical Conduct in Human Research, this form of secondary data is consistent with Outcome A of the University of Sydney Research Ethics Board (S1 File) and does not require additional ethics committee approval from the University.

## Results

In this study, a total of 1735 mother-child dyads were included (Table 2). The mean maternal age at birth of the study child was 35.6 years (SD = ±4.75); nearly 13% (n = 221) of mothers smoked during their pregnancy; approximately 31% (n = 544) of mothers consumed alcohol during pregnancy, about 5% (n = 87) of mothers had gestational diabetes, 7% (n = 118) reported hypertension during pregnancy, and 14% (n = 248) reported having post-natal depression. Table 2 also shows that more than half of the children were girls (n = 890, 51.3%); the majority were from major cities (n = 1240, 71.5%); and most respondents were from higher socio-economic status (advantaged income quintiles (n = 1248, 72%—a combination of quintile 3, 4 and 5). About 86% (n = 1495) were born at term, and around 93% (n = 1627) had normal birth weight.

**Table 2:** Descriptive statistics (n = 1735).

Characteristics		Total	ARTs used by the mother of the study child	ARTs not used by the mother of the study child
		n (%)	n (%)	n (%)
Total		1735 (100.0)	89 (5.1)	1646 (94.9)
Sex of the study child	Boys	845 (48.7)	43 (5.1)	802 (94.9)
	Girls	890 (51.3)	46 (5.2)	844 (94.8)
Maternal age^1^		Mean = 35.6; SD = 4.75	Mean = 37.1; SD = 4.73	Mean = 35.4; SD = 4.72
Smoked during pregnancy	No	1514 (87.3)	80 (5.3)	1434 (94.7)
	Yes	221 (12.7)	9 (4.1)	212 (95.9)
Drank alcohol during pregnancy	No	1191 (68.7)	69 (5.8)	1122 (94.2)
	Yes	544 (31.4)	20 (3.7)	524 (96.3)
Diabetes during pregnancy	No	1648 (95.0)	80 (4.9)	1568 (95.1)
	Yes	87 (5.0)	9 (10.3)	78 (89.7)
Hypertension (HTN) during pregnancy	No	1617 (93.2)	85 (5.3)	1532 (94.7)
	Yes	118 (6.8)	4 (3.4)	114 (96.6)
Postnatal depression after pregnancy	No	1487 (85.7)	80 (5.4)	1407 (94.6)
	Yes	248 (14.3)	9 (3.6)	239 (96.4)
Mother’s employment status during pregnancy	Employed	1192 (68.7)	68 (5.7)	1124 (94.3)
	Unemployed	543 (31.3)	21 (3.9)	522 (96.1)
Gestational age	Term	1495 (86.2)	70 (4.7)	1425 (95.3)
	Preterm	120 (6.9)	14 (11.7)	106 (88.3)
	Post-term	121 (6.9)	5 (4.2)	115 (95.8)
Child’s birth weight	Normal	1627 (93.8)	73 (4.5)	1554 (95.5)
	Low birth weight	108 (6.2)	16 (14.8)	92 (85.2)
Area of residence	Major cities	1240 (71.5)	73 (5.9)	1167 (94.1)
	Regional/Remote	495 (28.5)	16 (3.2)	479 (96.8)
IRSAD quintiles	Quintile 1	207 (11.9)	7 (3.4)	200 (96.6)
	Quintile 2	280 (16.1)	13 (4.6)	267 (95.4)
	Quintile 3	329 (19.0)	16 (4.9)	313 (95.1)
	Quintile 4	401 (23.1)	21 (5.2)	380 (94.8)
	Quintile 5	518 (29.9)	32 (6.2)	486 (93.8)

^1^ Continuous variable

^2^ Gestational age: Term (37–41 weeks of pregnancy), preterm (20–36 weeks of pregnancy), and post-term pregnancy (>42 weeks)

^3^ Child birthweight: Normal (birthweight between 2500 and <4500 grams), and low birth weight (birthweight <2500 grams)

In addition, Table 2 illustrates that 5.1% (n = 89) of the mothers used ARTs for conceiving the study child. The mean maternal age was slightly higher for those mothers who used ARTs compared to those mothers who did not use ARTs (Mean 37.1 vs 35.4 years old) for conceiving the study child.

The proportion of mental disorders in the study population was 22.1% (n = 384), as reported in Table 3. Among the mothers who used ARTs for pregnancy, the percentages of children with or without mental disorders were similar, around 5% (Table 3).

**Table 3:** Mental disorders in the study child (n = 1735), and stratified by the use of ARTs by the mother of the study child.

	No, n (%)	Yes, n (%)
Mental disorders (n = 1735)	1351 (77.9)	384 (22.1)
ARTs not used by the mother of the study child	1281 (94.8)	365 (95.1)
ARTs used by the mother of the study child	70 (5.2)	19 (5.0)

The longitudinal analysis (logistic regression models) determining the impact of ARTs used by mothers on their offspring’s mental health is shown in Table 4. The association between the use of ARTs by mothers to obtain pregnancy and mental disorders in their children was not statistically significant in both unadjusted and adjusted models. The adjusted model reveals that maternal smoking during pregnancy (OR: 1.79, p = 0.003, 95% CI: 1.22–2.63), postnatal depression (OR: 2.19, p<0.001, 95% CI: 1.55–3.11), and maternal unemployment during pregnancy (OR: 1.68, p<0.001, 95% CI: 1.26–2.24) increased the likelihood of their offspring having mental disorders compared to their respective counterparts. Girls were 1.67 times (p<0.001, 95% CI: 1.25–2.21) more likely to report mental disorder compared to boys; and children born with low birthweight (OR: 1.75, p<0.044, 95% CI: 1.01–3.04) was statistically significantly associated with the increased probability of developing mental disorders compared to those who were born with normal weight. While the presence of gestational diabetes in mothers decreased the risk of developing poor mental health in children compared to those mothers who did not have diabetes during pregnancy.

**Table 4:** Impact of using ARTs by mothers on their children’s mental health taking into account several potential confounders.

		Mental disorders				
		Unadjusted OR	p-value (95% CI)	Adjusted OR (95% CI)	p-value (95% CI)	VIF^1^
History of using ARTs by mother	No	Ref.		Ref.		
	Yes	1.06	0.843 (0.58–1.94)	1.21	0.534 (0.65–2.23)	1.02
Maternal age		0.97*	0.034 (0.93–0.99)	0.98	0.147 (0.95–1.00)	1.03
Smoked during pregnancy	No	Ref.		Ref.		
	Yes	2.16***	0.000 (1.49–3.14)	1.79**	0.003 (1.22–2.63)	1.03
Drank alcohol during pregnancy	No	Ref.		-		
	Yes	1.04	0.777 (0.77–1.40)			
Diabetes during pregnancy	No	Ref.		Ref.		
	Yes	0.41**	0.007 (0.22, 0.78)	0.45*	0.021 (0.23–0.88)	1.01
Hypertension (HTN) during pregnancy	No	Ref.		-		
	Yes	1.25	0.450 (0.69–2.25)			
Postnatal depression after pregnancy	No	Ref.		Ref.		
	Yes	2.31***	0.000 (1.64–3.25)	2.19***	0.000 (1.55–3.11)	1.04
Mother’s employment status during pregnancy	Employed	Ref.		Ref.		
	Unemployed	1.76***	0.000 (1.33–2.34)	1.68***	0.000 (1.26–2.24)	1.01
Gestational age	Term	Ref.		-		
	Preterm	1.26	0.383 (0.74–2.12)			
	Post-term	0.61	0.057 (0.35–1.01)			
Child’s birth weight	Normal	Ref.		Ref.		
	Low birth weight	1.87*	0.022 (1.09–3.17)	1.75*	0.044 (1.01–3.04)	1.02
Child’s sex	Boys	Ref.		Ref.		
	Girls	1.49**	0.004 (1.13–1.97)	1.67***	0.000 (1.25–2.21)	1.01
Area of residence	Major cities	Ref.		-		
	Regional/Remote	1.16	0.322 (0.86–1.59)			
IRSAD quintiles	Quintile 1	Ref.		-		
	Quintile 2	0.99	0.980 (0.61–1.62)			
	Quintile 3	0.79	0.345 (0.48–1.28)			
	Quintile 4	0.82	0.399 (0.51–1.30)			
	Quintile 5	0.68	0.089 (0.45–1.05)			
Model performance tests						
Goodness-of-fit tests^2^	-			0.3373		
Link test^3^	-			3.16***		
Mean VIF (Max.)	-			1.03 (1.04)		

OR: Odds ratio; CI: confidence interval; Level of significance:

*p<0.001,

**p<0.01 and

***p<0.05;

^1^ VIF (Variance Inflation Factor): an indicator of measuring multicollinearity; as a rule of thumb, VIF>10 indicates a high correlation and VIF around 1 indicates no such correlation and regression can be conducted;

^2^ Goodness-of-fit test: A p-value of <0.05 indicates poor fit and a p-value closer to 1 indicates a good logistic regression model fit;

^3^ Link test (Model specification test): hat of the variable of prediction (i.e. mental disorder in this model) for the tested model should be significant (p<0.05) to specify the model correctly.

**Fig 2:**
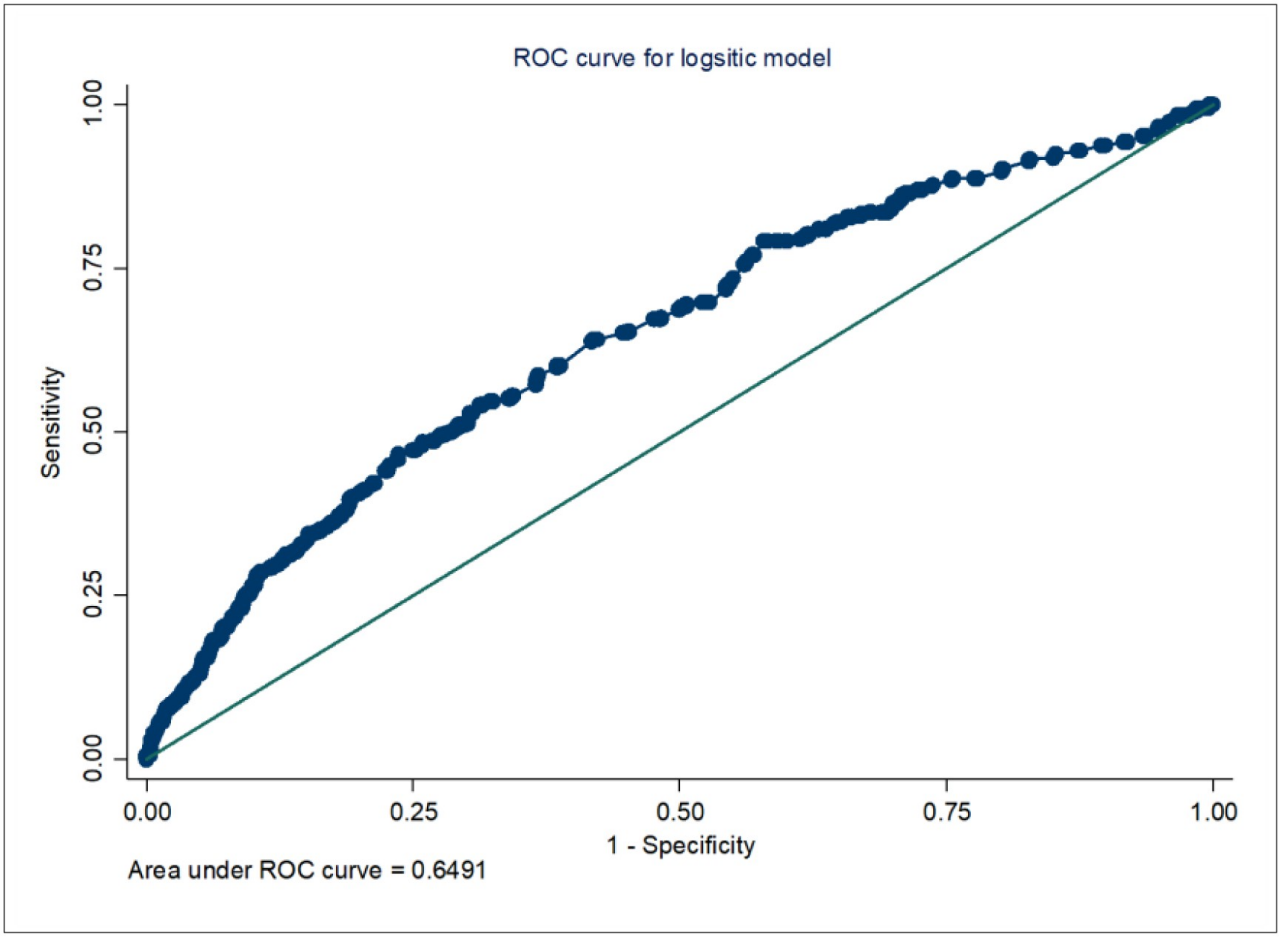
ROC curve analysis—A post hoc analytic procedure to logistic regression.

Furthermore, Table 4 depicts the results obtained from several model performance tests. For instance, the Goodness-of-fit tests revealed no significant difference exists between the observed data and the model (p>0.05), suggesting well-fitted models. In addition, the Link test confirmed that the model was specified correctly (i.e. hat of the variable of prediction for the tested model was significant at p<0.000), and the VIF with a mean of 1.03 showed no evidence of multicollinearity among predictor variables. Lastly, the area under ROC curves (more than 0.50) confirmed the satisfactory predictive power of the model (Fig 2).

## Discussion

The results of this study are encouraging for the parents of children conceived through ARTs, suggesting that their offspring do not have deleterious mental health outcomes as young adults compared to their spontaneously conceived peers. Notably, although this study aspired to control for sociological and obstetric confounders, there were no adverse effects of ARTs on children’s mental health regardless of whether these covariates were excluded or included in the regression model. Nevertheless, we found that several confounding variables did affect childhood mental health. The children of mothers who smoked during pregnancy, mothers with postnatal depression and mothers who were unemployed during pregnancy were more likely to have children with mental disorders compared to their respective counterparts. Moreover, our study revealed that girls (vs boys) and being born with low birthweight (vs normal birthweight) were more likely to develop poor mental health. Maternal gestational diabetes was found to be significantly associated with a decreased risk of developing mental disorders in children compared to those mothers who did not have diabetes during pregnancy.

Although there is significant controversy within the present literature around the relationship between ART use by mothers and mental disorders in their offspring, this null result adds further weight to the body of studies that have suggested that there is no significant relationship [19–21, 24–26] and draws doubt on studies that have postulated a deleterious relationship [26–34]. This study also adds credence to the established notion that children conceived through ARTs such as IVF and other techniques are indistinct from their peers as adults in terms of mental well-being [13, 14, 32]. In terms of an underlying mechanism for the deleterious impact observed in other studies on the effect of ART use on children’s mental health, there are multiple plausible explanations. The well-established risk of low birth weight and preterm birth among children conceived through IVF and the subsequent negative impact of this on neurodevelopment might partially explain these results [9, 11]. Other studies have suggested that microenvironmental differences in zygotes conceived through ART compared to those conceived spontaneously produce long-lasting developmental and epigenetic consequences, which may underlie an increased childhood risk of mental disorders [39, 40]. Nevertheless, these epigenetic consequences are known to be modest and normalised by adulthood [15, 16]. It is possible that these epigenetic differences may underlie the time-dependent effect observed in one study, further underscoring the importance of studying populations at multiple time intervals [32].

Furthermore, this study also provided interesting insights into the nature and strength of confounding sociological and obstetric covariates. For instance, our study revealed that maternal smoking during pregnancy increases the risk of poor mental health in children, and this finding is supported by the previous literature [41]. Another cohort study claimed that maternal smoking during pregnancy can be associated with the development of mental disorders in children depending on the number of cigarettes consumed by mothers per day [42]. Conversely, a few studies have found no association between maternal smoking during pregnancy and the risk of developing poor mental health in offspring [43, 44]. In addition, the current study also found that postnatal depression in mothers was significantly associated with the increased likelihood of developing mental disorders in their offspring, which is consistent with previous research findings [45, 46]. While another study reported that persistent postpartum depression is associated with children’s internalising problems but not with externalising mental health problems [47]. A meta-analysis published in the year 2020 suggests the need for future research to focus on exploring the neurobiological and pathophysiological mechanisms of the association between postnatal depression in mothers and mental health disorders in their children [45]. In addition, our study found that children born with low birthweight were more likely to develop mental disorders in adolescence and early adulthood compared to those born with normal birthweight, which has been corroborated by a recent meta-analysis [48]. However, evidence from a systematic review demonstrates that evidence behind the association between low birthweight children and the development of mental disorders in adulthood is uncertain [49]. Moreover, consistent with past research, the current study found that girls were more likely to report mental disorders compared to boys [50, 51].

Even though the current study utilized nationally representative data of Australian children, and the longitudinal cohort study design is an asset, providing converging evidence for the lack of impact of ART use by the mothers on the mental health of their offspring during adolescence/early adulthood, the study has some limitations. For instance, our study may suffer from selection bias due to non-responses. A further limitation of our study is the self-report nature of mental health, which may carry the risk of social desirability bias. Moreover, our study did not find a statistically significant impact (positive/negative) of using ARTs by mothers on mental health in their offspring may be due to the small size, resulting over/underestimation, and a future study with a greater sample size might be able to get a statistically significant result measuring the same effect. Furthermore, we were not able to include details on some variables such as the types of ART used by the mothers, frequency/amount of alcohol consumption by mothers and frequency of smoking by mothers during pregnancy—as these data were not collected from participants.

In conclusion, this study identified that the use of ARTs by mothers did not affect the mental health of their offspring during adolescence or early adulthood. The results of this study should contribute to the broader literature which can likely reassure parents considering ARTs that their children stand to develop into healthy adults both psychologically and neurodevelopmentally. These findings also reiterate the importance of counselling expectant mothers to avoid smoking during pregnancy and also the value of high-quality antenatal and perinatal care.

## Supporting information

S1 File Ethics guidelines for USYD researchers. (PDF)
